# Supplementary material for: Factors Associated with COVID-19 Vaccine Booster Hesitancy: A Retrospective Cohort Study, Fukushima Vaccination Community Survey
Source: Vaccines (Basel). 2022 Mar 26;10(4):515. doi: 10.3390/vaccines10040515 (PMC9032295; doi:10.3390/vaccines10040515)
Supplement: Supplementary file 1 [file vaccines-10-00515-s001.zip › vaccines-1629101-supplementary.pdf]

Supplementary Table S1. A logistic regression to identify variables associated with vaccine avoidance

	B (se)	OR (95% CI)	*p*-Value
Age	−0.200	0.98 (0.96–1.00)	0.034
Sex (base: male)	0.117	1.12 (0.63–2.01)	0.70
**Adverse reaction at 2nd dose**			
37.5 °C fever	−0.032	0.97 (0.49–1.91)	0.93
fatigue	−0.486	0.62 (0.32–1.18)	0.142
headache	0.267	1.31 (0.66–2.59)	0.45
joint pain	−0.163	0.85 (0.45–1.61)	0.62
nausea	1.423	4.15 (1.82–9.47)	0.001
IgG antibody titer	0.958	2.61 (1.31–5.18)	0.006
